# Evaluation of the Antidiabetic Activity of Hydromethanolic Roots Extracts of *Rumex abyssinicus* Jacq: (Polygonaceae) in *Swiss* Albino Mice

**DOI:** 10.1155/2022/5193250

**Published:** 2022-06-30

**Authors:** Baye Yrga Adugna, Getinet Mequanint Adinew, Kefyalew Ayalew Getahun, Abyot Endale Gurmu, Alemshet Yirga Berhie, Tewachew Awoke, Getaye Tessema Desta

**Affiliations:** ^1^Department of Pharmacy, Bahir Dar Health Science College, Bahir Dar, Ethiopia; ^2^Department of Pharmacology, School of Pharmacy, College of Medicine and Health Sciences, University of Gondar, Gondar, Ethiopia; ^3^Department of Pharmacognosy, School of Pharmacy, College of Medicine and Health Sciences, University of Gondar, Gondar, Ethiopia; ^4^Department of Adult Health Nursing, School of Health Science, College of Medicine and Health Science, Bahir Dar University, Bahir Dar, Ethiopia; ^5^Department of Medical Laboratory Sciences, College of Medicine and Health Sciences, Bahir Dar University, Bahir Dar, Ethiopia; ^6^Department of Pharmacy, College of Medicine and Health Sciences, Debre Tabor University, Debre Tabor, Ethiopia

## Abstract

**Introduction:**

Diabetes Mellitus isa chronic metabolic disorder that required long-lasting treatment. In Ethiopian traditional medicine practices, abundant plants have been used for the treatment of diabetes mellitus for a long period. The root of *Rumex abyssinicus* is employed for the treatment of diabetes mellitus by Ethiopians. This study aimed to investigate the antidiabetic activity of the crude extract of *Rumex abyssinicus* root in *Swiss* albino mice.

**Methods:**

Cold maceration technique and hydro methanolic (80% methanol) solvent with occasionally shaking were employed during the crude extraction processes. To evaluate the antidiabetic activity of the crude extract, normoglycemic, glucose-loaded, and streptozotocin-induced diabetic models were used. In each model, the overnight fasted mice were randomly divided into five groups for normoglycemic and glucose-loaded models as a negative control, positive control, and three tested groups, whereas, in streptozotocin-induced diabetic models, the mice were grouped into six groups like one diabetic and one normal negative control groups, three diabetic tested groups, and one diabetic positive group. Each group comprised six mice. For all models, the tested groups received the crude extract at 100, 200, and 400 mg/kg doses, both diabetic and nondiabetic negative control groups received 10 ml/kg distilled water, and positive groups received 5 mg/kg glibenclamide.

**Results:**

The crude extract of the plant did not show any sign of toxicity up to 2000 mg/kg dose. In normoglycemic and oral glucose tolerance tests, the crude extract significantly (*p* < 0.01) reduced the blood glucose level at 200 and 400 mg/kg doses. In the streptozotocin-induced diabetes models, a significant effect was observed at all tested doses.

**Conclusion:**

The finding of this study revealed that the crude extract of the plant owned antidiabetic activity and supports the traditional use of *Rumex abyssinicus* root for the treatment of diabetes mellitus.

## 1. Introduction

Diabetes mellitus (DM) is a metabolic disorder of carbohydrates, proteins, and fats. It characterizes by persistent hyperglycemia and develops as a consequence of a defect in insulin secretion or impairment of its action [1–3]. The signs and symptoms of DM include polyuria, polyphagia, polydipsia, calorie loss, generalized weakness, poor wound healing, numbness, blurry vision, and weight loss. The secondary complication of DM includes retinopathy, vascular complications, peripheral neuropathy, renal failure, vascular complications, and limb amputations [4]. Now a day, DM is a serious health issue for both developed and developing countries. Based on the estimation of the International Diabetes Federation (IDF) 2021, the global prevalence of DM was 10.5% (536.6 million people) in 20–79 years old. By 2045, this figure is expected to be 12.2% (783.2 million) by a 46% increment. In 2021, the prevalence of DM was higher in urban areas (12.1%) as compared to the rural area (8.3%), in high-income (11.1%) countries than in low-income countries (5.5%). During this year, the global expenditure for DM was 966 billion United States Dollars (USD) and is expected to be raised by 1,054 billion USD by 2045 [5]. In Ethiopia, 1.3 million people (20–79 years old) lived with DM and the prevalence was 2.9%. This figure is projected to be 1.8 million by 2030 [6].

The impact of DM is still a challenge for humankind. It impairs the quality of life and decreases the daily income of individuals and the development of countries [7]. Currently, numerous antidiabetic drugs are available on the market, but there was no satisfactory antidiabetic drug for all diabetic patients. They are not easily accessible at an affordable price and cause dangerous side effects like weight gain and hypoglycemia with sulfonylureas, lactic acidosis, metallic taste, and weight loss in metformin [8]. Besides this, no antidiabetic drugs produce a curative effect [9]. Consequently, looking and searching for safe and effective antidiabetic drugs become a day-to-day issue in the pharmaceutical science and medical world. Across the world, the majority of people still depend on the use of plants for the treatment of DM and its complication [10]. The reason for this dependency include easily accessible of plants with affordable price, ease for self-administration and use, people's belief that plants have fewer side effects, and they have high cultural acceptance [6]. *Rumex abyssinicus* (*R. abyssinicus*) is categorized under a family of Polygonaceae and distributed in tropical Africa, particularly in Central and Eastern Africa [11]. In Ethiopia's folklore medicine, *R. abyssinicus* has a great role in the treatment of different human ailments. Traditionally, *R. abyssinicus* is used for the treatment of DM, and hepatitis [12]. The root and the stem were used for the treatment of diabetes, hypertension, wounds, cancer [13, 14], *tuberculosis*, teeth infections, abdominal problems, stomach pain [15, 16], headache and toothache [17], asthma, liver problems, common cold, inflammation, and painful conditions [12]. The leaf part is also used for the treatment of ascariasis and headache [17], and malaria [18].

Several *in vitro* and *in vivo* studies have been conducted on different parts of *R. abyssinicus.* The root extract exhibited good antioxidant [19], antibacterial and chemopreventive potential on preneoplastic lesions [20], antibacterial activity on *Streptococcus* pyogenes and *Staphylococcus aureus*, and antiviral against Coxsackie virus B3 and influenza A virus, and anti-inflammatory activities [21]. The methanolic extract of the rhizomes showed diuretic and analgesic activity [22], wound healing and anti-inflammatory activity [11], and hepatoprotective and radical scavenging activities while the leaves extract demonstrated anti-malarial activity [23]. The methanolic extract of bulbs of *R.abyssinicus* also revealed antihelminthic activity [24]. Furthermore, some of the *Rumex* species also showed a glucose-lowering activity. The ethanolic root extract of *R.nepalensis* demonstrated *in vitro α*-amylase inhibition, *in vivo* hypoglycemic activity in normal and glucose-loaded rats [25]. The phytochemical screening test on the root extract revealed the presence of saponins, flavonoids, tannins, steroids, anthraquinones, alkaloids, quinones, phenol, and glycosides, but the absence of terpenoids [26]. The folklore antidiabetic uses, *in vitro,* and *in vivo* studies provide significant clues to conducting this experiment. Moreover, until this time, the antidiabetic activity of the root extract of *R.abyssinicus* was not conducted. Therefore, this study aimed to evaluate the safety and antidiabetic efficacy of hydro methanolic root extract of *R.abyssinicus* in streptozotocin-induced diabetic mice.

## 2. Materials and Methods

### 2.1. Materials, Drugs, and Chemicals

The following materials, drugs, and chemicals were used during the study. Methanol absolute (Nice Chemicals, India), streptozotocin (Sigma- Aldrich, Germany), glibenclamide (Julphar Pharmaceuticals, Ras Al Khaimah, UAE), citric acid monohydrate (Lab Tech Chemicals, India), trisodium citrate dihydrate (Blulux Laboratories, India), distilled water, 40% glucose solution (Reyoung Pharmaceuticals, China), Wagner's reagent, Mayer's reagent (May and Baker LTD Dagenham, England), ferric chloride solution, lead acetate, chloroform (Sd Fine Chem. Limited T.V Industrial Estate, India), sulfuric acid (Fisher Scientific, UK), and sodium hydroxide (BDH, chemical lab, England).

### 2.2. Plant Material

The fresh root of *R.abyssinicus* was collected from the South Gondar Zone of the Amhara region, Northwest Ethiopia, and wrapped with plastic sheets during transportation. The plant was identified and authenticated by a Botanist at the University of Gondar and the voucher number ED001/2011 was deposited in the Herbarium of Biology Department, Faculty of Natural and Computational Science, University of Gondar.

### 2.3. Methods

#### 2.3.1. Preparation of Plant Extract

The root of *R.abyssinicus* was thoroughly washed with tap water to remove dirt and derbies. Then, the root was dried under a shadow in the pharmacy laboratory room. After drying, the root of the plant was subjected to size reduction using a mechanical miller. A mass of 1.6 kg coarse powdered was macerated in a sufficient amount of 80% methanol for 72 h with occasional stirring and agitation. Following 72 h maceration, the extract was separated from the mark using filter paper (Whatman No.1). The marc was re-macerated two times with fresh 80% methanol. The filtrates obtained from the three successive filtrations were combined and concentrated using Rotary Evaporator (Yamato, Japan) set at 40°C. Finally, the semi-dried residues were frozen in the refrigerator overnight and then, lyophilized to completely dry using a lyophilizer (Lab freeze, China) [27]. Finally, 188.8 g (11.8%) of a dark red powder of crude extract was obtained. The obtained crude extract was stored in an airtight bottle in the refrigerator until it was subjected to the experimental procedure.

#### 2.3.2. Experimental Animals

To conduct this experiment, 6–8 weeks of age and 20–30 g body mass of *Swiss* albino male mice were used for all experimental models (oral glucose loaded, normoglycemic, and STZ-induced diabetic models). Relative to male mice, female mice are more resistant to developing DM secondary to STZ administration [28, 29]. Thereby, the female mice were excluded from this study. The mice were gained from the animal house of the Department of Pharmacology, School of Pharmacy, and the University of Gondar. The mice dwelled in a clean metabolic cage that was bedded with a chip of wood under a standard condition (12 h light and 12 h dark cycle) with free access to a commercial pellet diet, which was purchased from the Ethiopian Public Health Institution (EPHI), Addis Ababa, Ethiopia, and water ad libitum. Before initiation of the experiment, the mice were acclimatized to the laboratory condition for seven days. The mice were handled according to the international standard guidelines set for the Care and Use of Laboratory Animals [30]. The ethical approval permission was requested and gained from the research and Ethics Committee of the Department of Pharmacology, the University of Gondar with ethical license number sopy/2311.

#### 2.3.3. Grouping and Dosing of Animals

For normoglycemic and oral glucose-loaded models, the overnight fasted mice were arbitrarily assembled into five categories with six mice in each category. Group I(negative control) was treated with 10 ml/kg of distilled water; Groups II–IV (the tested mice) and received 100, 200, and 400 mg/kg of the crude extract of plant respectively, and Group V (positive control) was treated with the standard drug, glibenclamide (GLC) 5 mg/kg based on the method illustrated by Belayneh *et al* [31]. In STZ-induced diabetic model, the overnight fasted mice were arbitrarily allocated into six groups with six mice in each group (five diabetic groups and one nondiabetic group). Group I and II were given 10 ml/kg of distilled water and served as normal negative control and diabetic negative control, respectively. Groups III–V (tested group) were administered the plant extract at doses of 100, 200, and 400 mg/kg respectively, whereas group VI (positive) was treated with the standard drug GLC 5 mg/kg [32]. The doses of the plant extract were determined based on the result of the acute oral toxicity test as explained by Desta *et al.* [33]. Accordingly, the middle dose was calculated as one-tenth of the limit dose of the plant extracted (200 mg/kg), and the lower and the higher doses were calculated as half and twice the middle dose (100 and 400 mg/kg) respectively. Each volume of the treatment doses was calculated based on Organization for Economic Cooperation and Development (OECD) guidelines that specified as 1 ml/100 g body weight of the rodents [34]. Since, the plant material is taken in the acclaimed area by oral route [14], the mice also received the doses of each treatment (crude extract of the plant, distilled water, and glibenclamide) through oral route using oral gavage. Both the crude extract of the plant and the standard drug GLC were dissolved in DW just before administration.

#### 2.3.4. Induction of Experimental Diabetes

Streptozotocin (STZ) is the most commonly used diabetogenic chemical to evaluate the antidiabetic activity of plant extract in experimental diabetes animal models. Intravenous or intraperitoneal (IP) high dose of STZ 100–200 mg/kg selectively destroyed the islet *β* cell of the pancreas by saving extrapancreatic cells of the mice and induced a rapid and permanent hyperglycinemia in mice [35, 36]. Before launching the experiment, STZ was dissolving in a 0.1 M citrate buffer (the pH was adjusted at 4.5) immediately before being administered to the experimental mice. The mice were fasted overnight (14 h) and injected freshly prepared STZ at a dose of 150 mg/kg via the IP route. Following 30 min of administration of STZ, the mice were permitted free access to both food and water. After 6 h administration of STZ, the mice were administered 1 ml/kg of 5% glucose solution for the next 24 h to prevent hypoglycemic shock and death secondary to massive and rapid *β*-cell necrosis that results in the release of large amounts of insulin. After three days of post-STZ administration, the mice were screened for diabetes. Mice with a fasted BGL greater than 200 mg/dl were incorporated in the study as diabetic mice [28, 37–39].

#### 2.3.5. Collection and Measuring of Blood Glucose Level

At the time of the experiment, the blood sample was taken aseptically from the tip of the fasted mice's tail after cutting with a surgical blade. The BGL was measured three times using a blood glucose meter and test strip. The measurement was performed three times and an average of the triplicates value was taken.

The percentage of blood glucose reduction was calculated based on the following formula [40].

(1) $$\begin{array}{c} \%\text{reduction in blood glucose level}=\frac{G_{0}-G_{p}}{G_{0}}\times 100. \end{array}$$

where *G*_0_ is blood glucose level at 0 h and *G*_*p*_ is post-treatment blood glucose level.

#### 2.3.6. Hypoglycemic Effects of the Crude Extract on Normoglycemic Mice

For the normoglycemic model, the mice fasted overnight (14 h) from food with free access to water. Then, the mice were grouped indiscriminately into five groups and treated as elucidated in the dosing and grouping section. Following grouping, the BGL was measured just before giving the treatment of each dose (at 0 h as baseline). After administration of the respective treatment dose, the blood sample was collected and BGL was determined at 1, 2, 3, and 4 h post-treatment. The procedure was done according to the method established by Belayneh, *et al* [31].

#### 2.3.7. Antihyperglycemic Effects of the Crude Extract on Oral Glucose Tolerance Tests in Normoglycemic Mice

The method for the determination of the effect of the crude extract on the oral glucose tolerance test was run based on the method utilized by Kifle *et al* [41]. The mice were grouped randomly and treated as described in the grouping and dosing section. The mice fasted from food overnight for 14 h and then the BGL was measured, as a baseline, just immediately before the treatment of the doses (at 0 h). Then, the mice received their respective treatment doses. After 0.5 h treatment; each mouse was loaded 2 g/kg glucose solution (40% w/v) orally. The blood sample was taken from the tip of each mouth tail aseptically and the BGL was measured at 0.5, 1, and 2 h post glucose administration. This procedure was done based on the methods established by Alene, Cherie Melaku *et al* [42].

#### 2.3.8. Antidiabetic Activity of Single Dose of Crude Extract on Streptozotocin-Induced Diabetic Mice

As depicted in the grouping and dosing section, overnight fasted (14 h) mice were grouped randomly into six groups with six mice per group. The mice received their respective doses as stated in the grouping and dosing section and the blood sample was collected. The BGL was measured immediately before treatment (0 h as a baseline), and 1, 2, 4, and 6 h after the respective treatment doses. The procedure followed the method described by Alene *et al* [43].

#### 2.3.9. Antidiabetic Activity of Repeated Daily Dose of Crude Extract on Streptozotocin-Induced Diabetic Mice

Following induction of diabetes by STZ, the mice were grouped into six groups (six mice in each group) as illustrated in the grouping and dosing part and the method for running the procedure was based on Alene *et al* [43]. The respective doses of the treatment (GLC, distilled water, and the tested doses of the crude extract) were provided to each experimental mouse daily for a total of 14 days. The overnight fasted (14 h) BGL was measured just before administration of the respective dose for each mouse (day 0) as a baseline, and then at the 7^th^ and 14^th^ days of post-treatment [44].

#### 2.3.10. Preliminary Phytochemical Screening

The crude extract of the *R. abyssinicus* root was subjected to a qualitative phytochemical screening test for the presence or absence of phenols, flavonoids, tannins, terpenoids, alkaloids, saponins, steroidal, and anthraquinones according to the following respective methods and procedures.

(i) Test for Phenols
    Ferric Chloride Test
    The crude extract (50 mg) was dissolved in 5 ml of distilled water and the 3 drops of 5% ferric chloride solution were added. The formation of a dark green/bluish-black color indicated the presence of phenols [45–47].
    Lead Acetate Test:
    The crude extract (50 mg) was dissolved in 5 ml of distilled water and the 3 ml of 10% lead acetate solution was added to this mixture. The formation of a white precipitate indicated the presence of phenols [46].
(ii) Test for Flavonoids
    Lead Acetate Test
    About 1 ml of the crude extract was mixed with a few drops of 10% lead acetate solution. The formation of yellow color precipitate showed the presence of flavonoids [46–48].
    Alkaline Reagent Test
    About 2 ml of the crude extract was mixed with 2 ml of 2% of sodium hydroxide solution in a test tube. The formation of an intense yellow color was turned colorless when 2 drops of dilute hydrochloric acid were added. This is an indication of the presence of flavonoids [46, 47, 49].
(iii) Test f'or Tannins
    Ferric Chloride Test
    To 1 mL of crude extract, an equal amount of ferric chloride solution was mixed. The formation of greenish-black color indicated the presence of tannins [50].
    Gelatin Test
    The crude extract was dissolved in 5 ml of distilled water. Then, 1% gelatin solution containing 10% sodium chloride was added to the crude extract. The formation of white precipitate indicated the presence of tannins [46, 47, 51].
(iv) Test for Alkaloids
    Mayer's Test
    About 50 gm of the crude extract was mixed with a few ml of diluted hydrochloric acid and then filtered. The filtrate was treated with 1–2 drops of Mayer's reagent (along the sides of the test tube). The formation of white creamy precipitate indicated the presence of alkaloids [46, 47].
    Wagner's Test
    About 50 gm of the crude extract was mixed with a few ml of diluted hydrochloric acid and then filtered. About 2 drops of Wagner's reagent were added to the filtrate (along the sides of the test tube). The appearance of brown/reddish precipitate indicated the presence of alkaloids [45, 47, 52].
(V) Test for Steroids
    Liebermann Burchard Test
    The crude extract (2 ml) was dissolved in an equal amount of chloroform. About 2 ml of concentrated sulfuric acid and 2 ml of acetic acid were added to the Mixture. The formation of green-colored precipitate indicated the presence of steroids [50, 53].
(VI) Test for Terpenoids
    Salkowski Test
    About 50 mg of the crude extract was dissolved in 5 ml of methanol and the extract was mixed with 2 ml of chloroform. The mixture was warmed and cooled. Then, 3 ml of concentrated sulfuric acid was added slowly along the sides of the test tubes. The formation of a radish brown-colored precipitation at the interface indicates the presence of terpenoids [54].
(VII) Test for Saponins
    Foam Test:
    The crude extract (50 mg) of the plant was diluted with DW up to 20 ml suspension. The suspension of the crude extract was shaken for 15 minutes. The presence of a 1 to 2 cm foam layer indicated the presence of saponins [45].
(VIII) Test for Anthraquinones
    Borntrager's Test
    The crude extract (6 g) was soaked in 10 ml of benzene for 10 minutes in a conical flask and the filtrate was filtrated. Then, 10 ml of 10% ammonia solution was added to the filtrate and shaken vigorously for 30 seconds. The observation of pink, violet, or red-colored in the ammonical solution or at the lower phase was an indication of the presence of anthraquinones [49].
    Ammonium hydroxide Test
    The crude extract **(**10 mg) was dissolved in isopropyl alcohol. One drop of concentrated ammonium hydroxide solution was added to the dissolved crude extract. The observation of a red color after 2 minutes indicated the presence of anthraquinone [49, 55].

#### 2.3.11. Acute Toxicity Study

An acute oral toxicity test was carried out according to OECD 2008 guideline 425 [56]. One female *Swiss* albino mouse fasted for 4 h on the first day of the test. Then, 2 g/kg of the extract was given orally and observed strictly for physical and behavioral changes for the next 24 h and special attention was given during the first 4 h. Based on the results from the first mouse, another four female mice were recruited and fasted for 4 h and then were given 2 g/kg as a single dose and observed for 14 days.

## 3. Statistical Analysis

Statistical Package for the Social Sciences (SPSS) Version 23 Software was used for statistical analysis. The results of the study were expressed as mean ± standard error of the mean. Means of all parameters among and within groups were compared using One-way Analysis of Variance (ANOVA) followed by Tuckey's post hoc multiple comparisons. The *p* -value <0.05 was considered statistically significant.

## 4. Results

### 4.1. Preliminary Phytochemical Screening

The qualitative phytochemical screening tests of the crude extract of the *R.abyssinicus* root illustrate the presence of phenols, flavonoids, tannins, steroids, saponins, alkaloids, anthraquinones, and terpenoids.  Table 1 presents the results of qualitative phytochemical screening tests of the crude extract of the root of R.abyssinicus.

### 4.2. Acute Oral Toxicity Study

After loading 2 g/kg of a single dose of the crude extract of *R. abyssinics* root, the mice were carefully observed within the first 24 h up to 14 days in their cage. During 14 day follow-up period, neither death nor any signs of toxicity such as a change in breathing rate, paw licking and shivering, restlessness, motor activity, and diarrhea were observed in any of the mice. This indicated that the median lethal dose (LD_50_) of the root extract, a dose requires to kill 50% of the experimental animal, is greater than 2 g/kg.

### 4.3. Hypoglycemic Effects of the Crude Extract on Normoglycemic Mice

The hypoglycemic effect of the crude extract of *R.abyssinics* root on normally overnight fasted mice presents in Table 2. There was no significant difference in fasting BGL across the group at baseline BGL. As compared to the negative control group, there was a significant effect on BGL in mice which received 200 (*p* < 0.01) and 400 mg/kg (*p* < 0.01) of the crude extract at 4^th^ h with 30.94% and 49.65% BGL reduction from the baseline, respectively, while the standard drug, glibenclamide, showed a significant reduction on the normal fasted BGL starting from at 1^st^ (*p* < 0.01), 2^nd^ (*p* < 0.001), 3^rd^ (*p* < 0.001), and 4^th^ h (*p* < 0.001). Mice received 100 mg/kg crude extract failed to show a significant reduction in BGL at all times.

### 4.4. Antihyperglycemic Effects of the Crude Extract on Oral Glucose Tolerance Tests in Normoglycemic Mice

The effect of crude extract of *R. abyssinicus* on oral glucose tolerance test in normoglycemic mice is presented in Table 3. The peak BGL was achieved within 0.5 h post-loading of 2 g/kg glucose to mice receiving distilled water. Mice received 400 mg/kg showed a significant reduction in BGL after 1 and 2 h loading of oral glucose, while 200 mg/kg at 2 h relative to mice received distilled water. Mice that received the standard drug (GLC 5 mg/kg) showed a significant reduction of the BGL at 0.5, 1, and 2 h after challenging the oral glucose as compared to the negative group.

### 4.5. Antihyperglycemic Activity of the Crude Extract on Single Dose in Streptozocin-Induced Diabetic Mice

All the tested crude extract doses of the root and glibenclamide 5 mg/kg showed a significant BGL (*p* < 0.001) reduction at any time as compared to the baseline BGL. Moreover, administration of crude extracts at all tested doses and glibenclamide 5 mg/kg significantly reduced the BGL (*p* < 0.001) as compared to the diabetic control (Table 4).

Results are expressed in mean ± SEM; *n* = 6 for each treatment. ^a^Compared to baseline fasting BGL, ^b^compared to diabetic control (DC), ^c^compared to RA 100 mg/kg, ^d^compared to RA 200 mg/kg, ^1^*p* < 0.05^2^*p* < 0.01, and ^3^*p* < 0.001, NC = negative control (received 10 ml/kg distilled water), DC = diabetic control (received 10 ml/kg distilled water), RA = *Rumex abyssinicus,* GLC = Glibenclamide, SEM = standard error of mean.

### 4.6. Antihyperglycemic Activity of Repeated Daily Doses of the Crude Extract in Streptozotocin-Induced Diabetic Mice

The outcome of the daily repeated doses of the crude extract of *R. abyssinicus* root on the BGL in diabetic and normal mice is summarized in Table 5. There was no significant discrepancy across the group on the BGL at the baseline. All three treatment doses of the root extract of *R. abyssinicus* revealed a significant (*p* < 0.001) reduction in the fasting BGLs on the 7^th^ and 14^th^ days as compared to the diabetic and normal control groups. The GLC treated group also showed a significant (*p* < 0.01) reduction in BGL on the 7^th^ and 14^th^ days when compared to the diabetic and normal control groups, but there was no a significant difference between GLC treated groups and groups treated with all the three root extracts of *R. abyssinicus*. There was no considerable discrepancy among the three doses of the crude extract treated groups.

Each value represents mean ± SEM; *n* = 6 for each treatment. ^a^compared to baseline, ^b^compared to diabetic control, ^c^ompared to GLC, ^n^compared to normal control, ^1^*p* < 0.05, ^2^*p* < 0.01, and ^3^*p* < 0.001. NC = negative control (received 10 ml/kg distilled water), DC = diabetic control (received 10 ml/kg distilled water), RA = *Rumex abyssinicus,* GLC = Glibenclamide, SEM = standard error of mean.

## 5. Discussion

Evaluation of acute oral toxicity test of the crude extract of *R. abyssynicus* verified that 2000 mg/kg dose did not show any sign of toxicity and death during 14 days of observation and confirmed that the folklore use of the root part is safe.

The animal model is an important tool to assess the pathophysiology mechanism of DM and the antidiabetic action of plant remedies. The induction of DM using STZ is a common one for the evaluation of the antidiabetic activity of medicinal plants in animal models [28, 57]. The chemical structure of STZ contains a glucose and methyl nitrosourea moiety. The glucose moiety guides the transport of STZ into the pancreatic *β*-cells, while the nitrosurea moiety causes the production of pancreatic *β*-cells toxicity. The *β*-cells of the islet take up STZ via glucose transporter 2 (GLUT2). The STZ selectively accumulated in pancreatic *β*-cells of islet and cells lacking this glucose transporter is resistant to STZ. Once inside *β*-cells, STZ causes cytotoxicity on *β*-cells via méthylation of deoxyribonucleic acid (DNA) strand and results in the fragmentation of the DNA. STZ also induces the activation of poly adenosine diphosphate ribosylation and nitric oxide release; thereby exerting oxidative stress through free radical generation. As a consequence, *β*-cells are destroyed and unable to secrete insulin which results in the development of DM hyperglycemia [28, 58, 59]. Previously studied research indicated that the pancreas contains stable (quiescent) *β*-cells which can restore themselves after being damaged by STZ and alloxan. This enhances insulin secretion and improves hyperglycemia [60, 61]. Plant extract from *Spondias pinnata* (Linn. f.) Kurz, *Coccinia grandis* (L.) Voigt and *Gmelina arborea* revealed the regeneration of *β*-cell in rats that developed diabetes secondary to alloxan [62].

In the evaluation of the hypoglycemic effect of *R. abyssinicus,* the crude extract of the root showed a significant dropping in the BGL in time and dose-dependent fashion in normoglycemic mice. This indicated that the root part of *R. abyssinicus* owned a hypoglycemic activity. This hypoglycemic effect was in line with the hypoglycemic effect of the root extract of *Rumex nepalensis*, in which the two plants are classified under the same family [25]. The oral glucose tolerance test is an important tool to assess the release of insulin and insulin resistance in clinical and research areas. The test is used to measure the ability of the body to use and clear glucose from the bloodstream and is used to diagnose pre-diabetes and diabetes cases [63]. In impaired glucose tolerance, the body required a long period to clear the challenged glucose. The impairment of glucose tolerance decreases the uptake of glucose by muscle, and fat, thereby the maintenance of blood glucose homeostasis is impaired [64]. Hence, the oral glucose tolerance test was done to evaluate the effect of the crude extract of *R. abyssinicus* on glucose homeostasis. Even though there was an increase in BGL after glucose loading from the baseline glucose level, 200 and 400 mg/kg crude extract and glibenclamide reduced significantly from peak level to baseline within 2 h compared with negative control. From previous studies, plant extracts lowered BGL by increasing insulin secretion, enhancing insulin sensitivity and glucose uptake at peripheral tissue, escalating glucose excretion, and inhibiting glycogenolysis, and gluconeogenesis [65, 66]. Nevertheless, the exact mechanism of action is unknown; the glucose-lowering action of the crude extract of *R. abyssinicus* root might be mediated via insulin secretion, enhancing insulin sensitivity and glucose uptake at peripheral tissue, escalating glucose excretion, inhibition of glycogenolysis and gluconeogenesis.

In STZ-induced diabetic mice, the crude extract of *R. abyssinicus* root showed a significant reduction in BGL as compared to diabetic control. The lower dose (RA 100 mg/kg) of the extract failed to show a reduction of BGL in normal and glucose-loaded animals, but there was a considerable reduction in BGL in STZ-induced diabetic mice. A comparable effect was also observed from the leaves of *Caylusea abyssinica* [37]. The finding of this study suggested that the lower dose of the crude extract was unable to overcome the physiological counter-regulatory mechanism. This inability might be due to the presence of low amounts and types of phytochemical constitutes of the crude extract of the root. The lower concentration of the phytochemical constitutes induces hypoglycemia in normoglycemic and glucose-loaded normal mice or the hypoglycemic nature of the lower dose would be apparent when there is an alteration in normal blood glucose regulatory systems by diabetes [37]. The reduction of BGL was in a dose and time-dependent manner. The preliminary phytochemical screening test of the crude extract of *R. abyssinicus* root showed the presence of phenols, flavonoids, tannins, saponins, anthraquinones, alkaloids, and terpenoids. Phytochemical constitutes are the responsible bioactive compounds for the antidiabetic activity of medicinal plants as reported previously studies [67, 68]. Saponin, isolated from *Tribulus terrestris L* extract, demonstrated a significant BGL reduction in alloxan-induced diabetic mice [69]. Alkaloids from the leaf extract of Catharanthus roseus (L.) G. Don, showed hypoglycemic activity *in vitro* study [70]. Rutin, is a flavonoid, that also demonstrated antidiabetic activity [71]. The antidiabetic mechanism action of these phytochemical constitutes mediates through reduction of glucose production in the liver, increase the expression of glucose transporter isoform 4 (GLUT4), enhancement of *β*-cell regeneration that increases the release of insulin, exerting an antioxidant effect, blocking of glucose absorption in the lumen of intestine, decreasing the activity of glucokinase enzyme [72–74].

The antihyperglycemic effect of *R. abyssinicus* root extract might be mediated through these phytochemical constituents either in an individually or synergistically action. Flavonoids, isolated from *Nelumbo nucifera* leaves, inhibited the activity of *α*-glucosidase, lipase, and *α*-amylase *in vitro* study [75]. Quercetin, which is a flavonoid, also showed a significant reduction in blood glucose levels in STZ-induced diabetic rats through regeneration of the pancreatic islets cell [76]. Alkaloids isolated from leaves of *Catharanthus roseus* (L.) G. Don demonstrated antioxidant and antidiabetic activities *in vitro* [70]. Tannin, which was presented in the crude extract of *R*.*abyssinicus* root, demonstrated antidiabetic activity by inhibiting alpha-amylase and alpha-glucosidase. Tannin also exerted its antidiabetic activity secondary to its antioxidant action on pancreatic *β*-cells [77]. Another study revealed that saponins from plant extract demonstrated antidiabetic activity in the alloxan-induced diabetic rats. An in-vitro study from previously studied indicated that alkaloids from *Catharanthus roseus* (L.) G. Don demonstrated antidiabetic activity through increasing glucose uptake [70]. The previous investigations revealed that the related species *Rumex vesicarius* showed significant antidiabetic activity in a dose-dependent fashion [78]. *Rumex rothschildianus*, another species under the family Polygonaceae, also showed antidiabetic activity *in vitro* study [79]. Since these two planets are in the same family and also support the observed antidiabetic activity of *R. abyssinicus* root extract. Although the exact antidiabetic mechanism action of *R. abyssinicus* is unknown, the possible antidiabetic mechanisms action of the *R.abyssinicus* root may be enhancements of insulin action at the target tissue, inhibition of carbohydrate absorption in small intestine, augmentation of insulin secretion in pancreatic *β*-cell as result of restoration of *β*-cell, and inhibition of gluconeogenesis.

## 6. Conclusion

The Hydromethanolic crude extract of the root of *R. abyssinicus* possesses significant glucose-lowering activity both in normal and STZ-induced diabetic mice in a dose and time-dependent manner. This study confirmed that the root part of *R. abyssinicus is* owned with antidiabetic activity and supported the folklore use of the root for the treatment of DM. The higher dose of the root part of the plant showed a significant blood glucose-reducing activity. Based on the acute oral toxicity study, the study plant showed a wide margin of safety.

## Figures and Tables

**Table 1:** The qualitative phytochemical screening of the crude extract of the root of *R. abyssinicus*.

Sample	Screened phytochemical	Types of test	The observed color	Result
1	Flavonoids	Lead acetate test	Yellow color precipitate	+
		Alkaline reagent test	Deep yellow color to colorless	+
		Ferric chloride test	Dark green/bluish-black color	+

2	Phenols	Lead acetate test	White precipitate	+
		Ferric chloride test	Greenish black color	+

3	Tannins	Gelatin test	White precipitate	+
		Mayer's test	White creamy precipitate	+
4	Alkaloids	Wagner's test	Brown/reddish precipitate	+
5	Steroids	Liebermann burchard	Green colored precipitate	+
6	Terpenoids	Salkowski test	Radish-brown precipitation	+

7	Saponins	Foam test	Foam layer	+
		Borntrager's test	Pink, violet, or red-colored	+
8	Anthraquinones	Ammonium hydroxide	Red color	+

+ = positive result.

**Table 2:** The effect of crude extract on blood glucose level in normoglycemic mice.

Treatment given	BGL in mg/dl				
	0 (h)	1	2 (h)	3	4 (h)
NC	117.17 ± 3.81	104.17 ± 4.63	128.00 ± 8.07	114.33 ± 8.59	105.87 ± 4.77
RA 100 (mg/kg)	130.17 ± 6.76	125.17 ± 18.36	116.50 ± 6.58	112.63 ± 17.98	103.33 ± 6.78
RA 200 (mg/kg)	117.33 ± 1.69	110 ± 5.32	105.50 ± 7.29	99.50 ± 4.58	81.03 ± 3.03^a2^
RA 400 (mg/kg)	119.17 ± 3.80	109.00 ± 1.46	101.83 ± 4.13	90.00 ± 2.98	60.00 ± 4.50^a3b3c1^
GLC 5 (mg/kg)	130.83 ± 1.25	78.33 ± 8.07^a2^	75.00 ± 7.04^a3b2c1^	72.17 ± 9.79^a1b1^	57.00 ± 1.44^a3b3c22^

Results are expressed in mean ± SEM, *n* = 6,^a^ compared to negative control,^b^ compared to RA 100 mg/kg,^c^ compared to RA 200 mg/kg,^d^ compared to RA 400 mg/kg, ^1^*p* < 0.05, ^2^*p* < 0.01, and ^3^*p* < 0.0011. RA = *R.abyssinicus*, DW = distilled water, NC = negative control (received 10 ml/kg distilled water, GLC = glibenclamide, SEM = standard error of mean.

**Table 3:** The effect of crude extract on glucose loaded in normoglycemic mice.

Treatment given	The BGL in mg/dl			
	0 (h)	0.5 (h)	1 (h)	2 (h)
NC	91.73 ± .60	194.40 ± 10.62	161.00 ± 8.62	134.00 ± 3.89
RA 100 (mg/kg)	93.87 ± 1.29	173.84 ± 23.21	151.80 ± 20.85	110.80 ± 13.87
RA 200 (mg/kg)	90.42 ± 1.58	152.97 ± 10.49	124.41 ± 5.33	93.53 ± 7.65^a1^
RA 400 (mg/kg)	91.64 ± 1.58	146.00 ± 1.29	108.64 ± 10.34^a1^	92.86 ± 9.15^a1^
GLC 5 (mg/kg)	93.200 ± 1.17	137.18 ± 9.03^a1^	80.52 ± 2.19^a3^	61.12 ± 4.68^a3b2^

Results are expressed in mean ± SEM; *n* = 6,^a^ compared to the negative control,^b^ compared to 100 mg/kg,^c^ compared to 200 mg/kg,^d^ compared to 400 mg/kg. ^1^*p* < 0.05, ^2^*p* < 0.01, and ^3^*p* < 0.001, NC = negative control (received 10 ml/kg distilled water), RA = *Rumex abyssinicus,* GLC = Glibenclamide, SEM = standard error of mean.

**Table 4:** Antidiabetic activity of single dose crude extract on streptozotocin-induced diabetic mice.

Group mg/kg	BGL (mg/dl)					% BGL reduction
	0 (h)	1 (h)	2 (h)	4 (h)	6 (h)	0 (h) to 6 (h)
NC	99.20 ± 1.97	96.00 ± 1.73	91.20 ± 1.62	95.2 ± 7.39	95.00 ± 3.31	4.2
DC	325.50 ± 1.25	332.50 ± 2.14	330.83 ± 2.64	337.00 ± 3.60	338.67 ± 1.80	-4.00
RA 100	324.10 ± 2.23	273.50 ± 1.45a1b3	242.00 ± 1.73a2b3	228.00 ± 4.21a3b3	216.33 ± 3.43a3b3	33.25
RA 200	325.00 ± 1.15	245.60 ± 1.30a2b3	215.60 ± 1.30a2b3	205.50 ± 1.26a3b3c2	194.50 ± 1.31a3b3c3	40.15
RA 400	326.60 ± 2.39	247.40 ± 1.70a2b3c3	199.40 ± .70a2b3c3d	178.83 ± 0.60a3b3c3d3	158.50 ± .96a3b3c3d3	51.72
GLC 5	325.80 ± 1.40	192.00 ± 1.15a3b3c3d3	165.80 ± 1.40a3b3c3d3	141.00 ± 1.91a3b3c3d3	124.83 ± 1.56a3b3c3d3	61.68

**Table 5:** Antihyperglycemic activity of repeated daily doses of the crude extract in streptozotocin-induced diabetic mice.

Experimental groups	The BGL in mg/dl			% BGL reduction	
	Day 0	Day 7	Day 14	Day 7	Day14
NC	96.40 ± 3.04	95.20 ± 7.38	97.80 ± 11.16	1.24	-1.45
DC	328.00 ± 2.11	325.17 ± 3.12^**n3**^	325.00 ± 4.35	0.86	0.91
RACE 100 (mg/kg)	323.83 ± 6.13	266.00 ± 10.32^**a2b2c3 n3**^	165.50 ± 10.70^**a3b3c1d3n3**^	17.86	48.89
RACE 200 (mg/kg)	333.33 ± 8.76	245.50 ± 7.03^**a3b3c3n3**^	142.80 ± 5.97^**a3b3d3n3**^	26.35	57.16
RACE 400 (mg/kg)	333.50 ± 7.95	231.17 ± 20.93^**a3b3c2n3**^	138.50 ± 11.71^**a3b3d3n3**^	30.65	58.47
GLC 5 (mg/kg)	330.67 ± 5.04	172.50 ± 8.18^**a3b3 n3**^	127.33 ± 3.27^**a3b3d3n3**^	47.83	61.49

## Data Availability

All the data that were used during the process of the experiment are available from the corresponding author and proved upon reasonable request.

## Abbreviations

BGL: Blood glucose level
DM: Diabetes mellitus
GLC: Glibenclamide
LD50: Median lethal dose
OECD: Organization for economic coperation and development
OGTT: Oral glucose tolerance test
STZ: Streptozotocin.

## References

[1] Okur M. E., Karantas I. D., Siafaka P. I. (2017). Diabetes Mellitus: a review on pathophysiology, current status of oral pathophysiology, current status of oral medications and future perspectives. *ACTA Pharmaceutica Sciencia*.

[2] Shewasinad A., Bhoumik D., Hishe H. Z., Masresha B. (2018). Antidiabetic activity of methanol extract and fractions of thymus schimperi ronniger leaves in normal and streptozotocin induce diabetic mice. *Iranian Journal of Pharmacology and Therapeutics*.

[3] Wadkar K., Magdum C., Patil S. S., Naikwade N. S. (2008). Anti-diabetic potential and Indian medicinal plants. *Journal of herbal medicine and toxicology*.

[4] Baynes H. W. (2015). Classification, pathophysiology, diagnosis and management of diabetes mellitus. *J diabetes metab*.

[5] Sun H., Saeedi P., Karuranga S. (2022). IDF diabetes atlas: global, regional and country-level diabetes prevalence estimates for 2021 and projections for 2045. *Diabetes Research and Clinical Practice*.

[6] Meresa A., Gemechu W., Basha H., Fekadu N. (2017). Herbal medicines for the management of diabetic mellitus in Ethiopia and Eretria including their phytochemical constituents. *AJADD*.

[7] Malviya N., Jain S., Malviya S. (2010). Antidiabetic potential of medicinal plants. *Acta Poloniae Pharmaceutica*.

[8] Mane P. B., Antre R. V., Oswal R. J. (2012). Antidiabetic drugs: an overview. *International Journal of Pharmaceutical and Chemical Sciences*.

[9] Kamboj A., Kumar S., Kumar V. (2013). Evaluation of antidiabetic activity of hydroalcoholic extract of cestrum nocturnum leaves in streptozotocin-induced diabetic rats. *Advances in Pharmacological Sciences*.

[10] Shukia R., Sharma S. B., Puri D., Prabhu K. M., Murthy P. S. (2000). Medicinal plants for treatment of diabetes mellitus. *Indian Journal of Clinical Biochemistry: Indian Journal of Clinical Biochemistry*.

[11] Mulisa E., Asres K., Engidawork E. (2015). Evaluation of wound healing and anti-inflammatory activity of the rhizomes of Rumex abyssinicus J. (Polygonaceae) in mice. *BMC Complementary and Alternative Medicine*.

[12] Suleman S., Alemu T. (2012). A survey on utilization of ethnomedicinal plants in Nekemte town, East Wellega (Oromia), Ethiopia. *Journal of Herbs, Spices, and Medicinal Plants*.

[13] Kloos H., Menberu T., Tadele A., Chanie T. (2014). Traditional medicines sold by vendors in Merkato, Addis Ababa: aspects of their utilization, trade, and changes between 1973 and 2014. *The Ethiopian Journal of Health Development*.

[14] Alemneh D. (2021). Ethnobotanical study of plants used for human ailments in Yilmana densa and Quarit districts of west Gojjam Zone, Amhara region, Ethiopia. *BioMed Research International*.

[15] Zenebe G., Zerihun M., Solomon Z. (2012). An ethnobotanical study of medicinal plants in Asgede Tsimbila district, Northwestern Tigray, northern Ethiopia. *Ethnobotany Research and Applications*.

[16] Vasas A., Orbán-Gyapai O., Hohmann J. (2015). The Genus Rumex: review of traditional uses, phytochemistry and pharmacology. *Journal of Ethnopharmacology*.

[17] Teklay A., Abera B., Giday M. (2013). An ethnobotanical study of medicinal plants used in Kilte Awulaelo District, Tigray Region of Ethiopia. *Journal of Ethnobiology and Ethnomedicine*.

[18] Karunamoorthi K., Sabesan S., Jegajeevanram K., Vijayalakshmi J. (2013). Role of traditional antimalarial plants in the battle against the global malaria burden. *Vector Borne and Zoonotic Diseases*.

[19] Mohammed S. A., Panda R. C., Madhan B., Demessie B. A. (2017). Extraction of bio-active compounds from Ethiopian plant material Rumex abyssinicus (mekmeko) root-A study on kinetics, optimization, antioxidant and antibacterial activity. *Journal of the Taiwan Institute of Chemical Engineers*.

[20] Girma B., Yimer G., Makonnen E. (2015). Effect of Rumex Abyssinicus on preneoplastic lesions in dimethylhydrazine induced colon carcinogenesis in rats. *BMC Complementary and Alternative Medicine*.

[21] Getie M., Gebre-Mariam T, Rietz R (2003). Evaluation of the anti-microbial and anti-inflammatory activities of the medicinal plants Dodonaea viscosa, Rumex nervosus and Rumex abyssinicus. *Fitoterapia*.

[22] Mekonnen T., Urga K., Engidawork E. (2010). Evaluation of the diuretic and analgesic activities of the rhizomes of Rumex abyssinicus Jacq in mice. *Journal of Ethnopharmacology*.

[23] Titanji V. P., Zofou D., Ngemenya M. N. (2008). The antimalarial potential of medicinal plants used for the treatment of malaria in Cameroonian folk medicine. *African Journal of Traditional, Complementary and Alternative Medicines: AJTCAM*.

[24] Tamokou J. D. D., Chouna J. R., Fischer-Fodor E. (2013). Anticancer and antimicrobial activities of some antioxidant-rich Cameroonian medicinal plants. *PLoS One*.

[25] Khatri D., Bahadur S., Chhetri S. B. B., Poudel P. (2018). Hypoglycemic and analgesic activity of root extract of rumex nepalensis. *World Journal of Pharmacy And Pharmaceutical Sciences*.

[26] Awoke S., Gedamu C. (2020). Chemical studies and antibacterial activity of the root of Rumex abyssinicus. *Earthline Journal of Chemical Sciences*.

[27] Sarker S. D., Latif Z., Alexander I. (2006). *Gray Natural Products Isolation*.

[28] Furman B. L. (2015). Streptozotocin‐induced diabetic models in mice and rats. *Current Protocols in Pharmacology*.

[29] Louet J.-F., LeMay C., Mauvais-Jarvis F. (2004). Antidiabetic actions of estrogen: insight from human and genetic mouse models. *Current Atherosclerosis Reports*.

[30] Council N. R. (2010). *Guide for the Care and Use of Laboratory Animals*.

[31] Belayneh Y. M., Birhanu Z., Birru E. M., Getenet G. (2019). Evaluation of in vivo antidiabetic, antidyslipidemic and in vitro antioxidant activities of hydromethanolic root extract of Datura stramonium L. (Solanaceae). *Journal of Experimental Pharmacology*.

[32] Manigauha A. (2018). Antioxidant and antidiabetic activity of isolated flavonoids from Alangium salvifolium leaves extracts. *International Journal of Green Pharmacy*.

[33] Desta G. T., Alemu M. A., Tsegaw A., Belete T. M., Adugna B. Y. (2021). Antidiarrheal effect of 80% methanol extract and fractions of clerodendrum myricoides (hochst.) vatke (lamiaceae) leaf in Swiss albino mice. *Evidence-based Complementary and Alternative Medicine*.

[34] OECD (2001). *OECD Guidelines For the Testing Of Chemicals–425*.

[35] Kottaisamy C. P. D., Raj D. S., Prasanth Kumar V., Sankaran U. (2021). Experimental animal models for diabetes and its related complications-a review. *Laboratory Animal Research*.

[36] Etuk E. (2010). Animals models for studying diabetes mellitus. *Agriculture and Biology Journal of North America*.

[37] Tamiru W., Engidawork E., Asres K. (2012). Evaluation of the effects of 80% methanolic leaf extract of Caylusea abyssinica (fresen.) fisch. & Mey. on glucose handling in normal, glucose loaded and diabetic rodents. *BMC Complementary and Alternative Medicine*.

[38] Baquer N. Z., Kumar P., Taha A., Kale R., Cowsik S., McLean P. (2011). Metabolic and molecular action of Trigonella foenum-graecum (fenugreek) and trace metals in experimental diabetic tissues. *Journal of Biosciences*.

[39] Kifle Z. D., Yesuf J. S., Atnafie S. A. (2020). Evaluation of in vitro and in vivo anti-diabetic, anti-hyperlipidemic and anti-oxidant activity of flower crude extract and solvent fractions of hagenia abyssinica (rosaceae). *Journal of Experimental Pharmacology*.

[40] Birru E. M., Abdelwuhab M., Shewamene Z. (2015). Effect of hydroalcoholic leaves extract of Indigofera spicata Forssk. on blood glucose level of normal, glucose loaded and diabetic rodents. *BMC Complementary and Alternative Medicine*.

[41] Kifle Z. D., Woldeyohanin A. E., Sema F. D. (2021). In vivo hypoglycemic, antihyperglycemic and antidyslipidemic effects of the solvent fractions of Hagenia abyssinica leaves in mice. *Metabolism Open*.

[42] Cherie Melaku B., Amare G. G. (2020). Evaluation of antidiabetic and antioxidant potential of hydromethanolic seed extract of datura stramonium linn (solanaceae). *Journal of Experimental Pharmacology*.

[43] Alene M., Abdelwuhab M., Belay A., Yazie T. S. (2020). Evaluation of antidiabetic activity of Ajuga integrifolia (Lamiaceae) root extract and solvent fractions in mice. *Evidence-based Complementary and Alternative Medicine*.

[44] Hammeso W. W., Emiru Y. K., Ayalew G. K., Kahaliw W. (2019). Antidiabetic and antihyperlipidemic activities of the leaf latex extract of Aloe megalacantha baker (Aloaceae) in streptozotocin-induced diabetic model. *Evidence-based Complementary and Alternative Medicine*.

[45] Banu K. S., Cathrine L. (2015). General techniques involved in phytochemical analysis. *International Journal of Advanced Research in Computer Science*.

[46] Shaikh J. R., Patil M. (2020). Qualitative tests for preliminary phytochemical screening: an overview. *International Journal of Chemical Studies*.

[47] Pandey A., Tripathi S. (2014). Concept of standardization, extraction and pre phytochemical screening strategies for herbal drug. *Journal of Pharmacognosy and Phytochemistry*.

[48] De Silva G. O., Abeysundara A. T., Aponso M. M. W. (2017). Extraction methods, qualitative and quantitative techniques for screening of phytochemicals from plants. *American Journal of Essential Oils and Natural Products*.

[49] Gul R., Jan S. U., Faridullah S., Sherani S., Jahan N. (2017). Preliminary phytochemical screening, quantitative analysis of alkaloids, and antioxidant activity of crude plant extracts from Ephedra intermedia indigenous to Balochistan. *The Scientific World Journal*.

[50] Shetty S., Vijayalaxmi K. (2012). Phytochemical investigation of extract/solvent fractions of Piper nigrum linn. Seeds and Piper betle linn. Leaves. *International Journal of Pharma Bio Sciences*.

[51] Bhatt S., Dhyani S. (2012). Preliminary phytochemical screening of Ailanthus excelsa Roxb. *International Journal of Current Pharmaceutical Research*.

[52] Sasidharan S., Chen Y., Saravanan D., Sundram K. M., Yoga Latha L. (2011). Extraction, isolation and characterization of bioactive compounds from plants’ extracts. *African Journal of Traditional, Complementary and Alternative Medicines*.

[53] Yadav R., Agarwala M. (2011). Phytochemical analysis of some medicinal plants. *Journal of Phytology*.

[54] Malik S., Ahmad M., Khan F. (2017). Qualtitative and quantitative estimation of terpenoid contents in some important plants of Punjab, Pakistan. *Pakistan Journal of Science*.

[55] Maria R., Shirley M., Xavier C. (2018). Preliminary phytochemical screening, total phenolic content and antibacterial activity of thirteen native species from Guayas province Ecuador. *Journal of King Saud University Science*.

[56] OECD (2008). *Acute Oral Toxicity: Up-And-Down Procedure*.

[57] Tegegne B. A., Mekuria A. B., Birru E. M. (2022). Evaluation of anti-diabetic and anti-hyperlipidemic activities of hydro-alcoholic crude extract of the shoot tips of Crinum abyssinicum hochst. Ex A. Rich (amaryllidaceae) in mice. *Journal of Experimental Pharmacology*.

[58] Lenzen S. (2008). The mechanisms of alloxan- and streptozotocin-induced diabetes. *Diabetologia*.

[59] Arora S., Ojha S. K., Vohora D. (2009). Characterisation of streptozotocin induced diabetes mellitus in swiss albino mice. *Global Journal of Pharmacology*.

[60] Oche O., Sani I., Chiaka N. G., Samuel N. U., Samuel A. (2014). Pancreatic islet regeneration and some liver biochemical parameters of leaf extracts of Vitex doniana in normal and streptozotocin-induced diabetic albino rats. *Asian Pacific Journal of Tropical Biomedicine*.

[61] El-Kordy E. A., Alshahrani A. M. (2015). Effect of genistein, a natural soy isoflavone, on pancreatic *β*-cells of streptozotocin-induced diabetic rats: histological and immunohistochemical study. *Journal of Microscopy and Ultrastructure*.

[62] Attanayake A. P., Jayatilaka K. A. P. W., Mudduwa L. K. B., Pathirana C. (2019). *β*-Cell regenerative potential of selected herbal extracts in alloxan induced diabetic rats. *Current Drug Discovery Technologies*.

[63] Bartoli E., Fra G. P., Schianca G. P. C. (2011). The oral glucose tolerance test (OGTT) revisited. *European Journal of Internal Medicine*.

[64] Benedé-Ubieto R., Estévez-Vázquez O., Ramadori P., Cubero F. J., Nevzorova Y. A. (2020). Guidelines and considerations for metabolic tolerance tests in mice. *Diabetes, Metabolic Syndrome and Obesity: Targets and Therapy*.

[65] Tesfaye A., Makonnen E., Gedamu S. (2016). Hypoglycemic and antihyperglycemic activity of aqueous extract of Justicia Schimperiana leaves in normal and streptozotocin-induced diabetic mice. *International Journal of Pharma Sciences and Research*.

[66] Pahlavani N., Roudi F., Zakerian M. (2019). Possible molecular mechanisms of glucose‐lowering activities of Momordica charantia (karela) in diabetes. *Journal of Cellular Biochemistry*.

[67] Kayarohanam S., Kavimani S. (2015). Current trends of plants having antidiabetic activity: a review. *Journal of Bioanalysis and Biomedicine*.

[68] Patel D., Kumar R., Laloo D., Hemalatha S. (2012). Diabetes mellitus: an overview on its pharmacological aspects and reported medicinal plants having antidiabetic activity. *Asian Pacific Journal of Tropical Biomedicine*.

[69] Li M., Qu W., Wang Y., Wan H., Tian C. (2002). Hypoglycemic effect of saponin from Tribulus terrestris. *Journal of Chinese medicinal materials*.

[70] Tiong S., Looi C., Hazni H. (2013). Antidiabetic and antioxidant properties of alkaloids from Catharanthus roseus (L.) G. Don. *Molecules*.

[71] Ghorbani A. (2017). Mechanisms of antidiabetic effects of flavonoid rutin. *Biomedicine & Pharmacotherapy*.

[72] Motlagh R. A., Mohebbi S., Moslemi M. (2019). Pancreatic *β*‐cell regeneration: from molecular mechanisms to therapy. *Journal of Cellular Biochemistry*.

[73] Chang C. L. T., Chen Y.-C., Chen H.-M., Yang N.-S., Yang W.-C. (2013). Natural cures for type 1 diabetes: a review of phytochemicals, biological actions, and clinical potential. *Current Medicinal Chemistry*.

[74] Aba P. E., Asuzu I. U. (2018). Mechanisms of actions of some bioactive anti-diabetic principles from phytochemicals of medicinal plants: a review. *Indian Journal of Natural Products and Resources*.

[75] Liu S., Li D., Huang B., Chen Y., Lu X., Wang Y. (2013). Inhibition of pancreatic lipase, *α*-glucosidase, *α*-amylase, and hypolipidemic effects of the total flavonoids from *Nelumbo nucifera* leaves. *Journal of Ethnopharmacology*.

[76] Vessal M., Hemmati M., Vasei M. (2003). Antidiabetic effects of quercetin in streptozocin-induced diabetic rats. *Comparative Biochemistry and Physiology—Part C: Toxicology and Pharmacology*.

[77] Serrano J., Puupponen-Pimiä R., Dauer A., Aura A.-M., Saura-Calixto F. (2009). Tannins: current knowledge of food sources, intake, bioavailability and biological effects. *Molecular Nutrition and Food Research*.

[78] Reddy N. S., Vidyasabbani fnm, Pravanthi B., Laxmi B. V., Harika B. (2016). Evaluation of antidiabetic activity of Rumex vesicarius in streptozotocin induced diabetic albino rats. *Research Journal of Pharmacology and Pharmacodynamics*.

[79] Jaradat N., Hawash M., Dass G. (2021). Phytochemical analysis, in-vitro anti-proliferative, anti-oxidant, anti-diabetic, and anti-obesity activities of Rumex rothschildianus Aarons. extracts. *BMC Complementary Medicine and Therapies*.

